# Classes of organic molecules targeted by a methanogenic microbial consortium grown on sedimentary rocks of various maturities

**DOI:** 10.3389/fmicb.2015.00589

**Published:** 2015-06-16

**Authors:** Margaux Meslé, Gilles Dromart, Frank Haeseler, Philippe M. Oger

**Affiliations:** ^1^Laboratoire de Géologie de Lyon, CNRS UMR 5276, Ecole Normale Supérieure de Lyon, Université de Lyon, LyonFrance; ^2^Tharsis-Energy, Ecole Normale Supérieure de Lyon, LyonFrance; ^3^Institut Français du Petrole Energies nouvelles, Rueil-MalmaisonFrance

**Keywords:** organic matter, maltenes, asphaltenes, kerogen, methanogenic consortia

## Abstract

Organic-rich shales are populated by methanogenic consortia that are able to degrade the fossilized organic matter into methane gas. To identify the organic fraction effectively degraded, we have sequentially depleted two types of organic-rich sedimentary rocks, shale, and coal, at two different maturities, by successive solvent extractions to remove the most soluble fractions (maltenes and asphaltenes) and isolate kerogen. We show the ability of the consortia to produce methane from all rock samples, including those containing the most refractory organic matter, i.e., the kerogen. Shales yielded higher methane production than lignite and coal. Mature rocks yielded more methane than immature rocks. Surprisingly, the efficiency of the consortia was not influenced by the removal of the easily biodegradable fractions contained in the maltenes and asphaltenes. This suggests that one of the limitations of organic matter degradation *in situ* may be the accessibility to the carbon and energy source. Indeed, bitumen has a colloidal structure that may prevent the microbial consortia from reaching the asphaltenes in the bulk rock. Solvent extractions might favor the access to asphaltenes and kerogen by modifying the spatial organization of the molecules in the rock matrix.

## Introduction

The microbial origin of methane accumulated in sedimentary basins was recently demonstrated by geochemical analyses based on the ^13^C values of methane collected from several gas plays around the world (Whiticar, 1999; Milkov, 2011). Microbial coalbed methane was detected in various basins in the USA, such as the Powder River (Green et al., 2008; Strapoc et al., 2010) and Illinois basins (Strapoc et al., 2007), and in Alaska (Jones et al., 2008; Penner et al., 2010; Strapoc et al., 2011). Field data also revealed the natural accumulation of biogenic methane in several organic-rich shales (Martini et al., 1996, 2003, 2008; Krumholz et al., 1997; Shurr and Ridgley, 2002; McIntosh et al., 2008; Schlegel et al., 2013) and oil reservoirs (Zengler et al., 1999; Aitken et al., 2004). Overall, biogenic gas is estimated to amount up to 20% of the total gas resource on our planet (Rice and Claypool, 1981). The microbial mineralization of organic molecules into methane in these environments, i.e., methanogenesis, relies on the syntrophic cooperation of different groups of microorganisms: fermenting and acetogenic bacteria, which degrade the complex molecules, and methanogenic archaea, which produce methane from the resulting simpler by-products (Schink, 1997; Zengler et al., 1999; Liu and Whitman, 2008; Morris et al., 2013).

Organic matter in sedimentary rocks is composed of a major insoluble fraction, i.e., kerogen, which represents usually from 80 to 99% of the OM; the rest being bitumen, the fraction of the OM soluble in organic solvents (Tissot et al., 1974; Tissot and Welte, 1984; Behar et al., 2008). Bitumen is divided into four sub-fractions: saturates (Atlas, 1981); aromatics which include monoaromatics such as benzene, toluene, ethylbenzene, and xylene (BTEX) as well as long chain alkylmonoaromatics and polycyclic aromatic hydrocarbons (PAHs); resins (heterocyclic NSO-compounds such as acids, bases, phenolics, and humic acids); and asphaltenes, a high molecular weight complex matrix (Vandenbroucke and Largeau, 2007). Depending on thermal maturity, aromatic, and saturated hydrocarbons can be less abundant than resins and asphaltenes in the bitumen of sedimentary rocks (Tissot et al., 1974; Chou et al., 1993), compared to the bitumen of oil in which the heavy fractions have undergone a thermal cracking, leading to a higher proportion of light fractions (Vandecasteele, 2005; Meslé et al., 2013b). Saturates, aromatics, resins, and asphaltenes are defined as the solvent-extractible fraction.

The recalcitrance of these organic compounds to biodegradation has been studied in oil reservoirs (Head et al., 2003; Peters et al., 2004) and coal (Orem et al., 2007; Wawrik et al., 2012). Most of the biodegradation processes in these environments occur under anaerobic conditions and lead to the formation of methane (Head et al., 2003; Jones et al., 2008). The highest biodegradation rates have been obtained on saturates, followed by the light aromatics (Walker et al., 1976; Leahy and Colwell, 1990; Jones et al., 2008), whereas high molecular weight aromatics and polar compounds (resins and asphaltenes) were considered relatively recalcitrant to biodegradation (Walker et al., 1976; Head et al., 2003; Gray et al., 2010; Haeseler et al., 2010). However, although this degradation pattern is widely accepted, a few exceptions were reported. Aromatic hydrocarbons were shown to be degraded prior to saturated hydrocarbons by marine microorganisms in a polluted harbor (Leahy and Colwell, 1990), as well as by microbial populations of coalbed methane plays (Formolo et al., 2008) in the Powder River and San Juan basins. This observation was explained by the apolar sigma bonds of saturates which make them less reactive than aromatics (Gray et al., 2010). Strict anaerobes have been reported to be able to grow on resins and asphaltenes as sole carbon and energy source (Magot et al., 2000), affecting the structure and composition of asphaltenes (Liao et al., 2009). The presence of methanogenesis metabolic intermediates (e.g., water-soluble aliphatic, cyclic, and aromatic hydrocarbons) within the extractable OM of coal formation waters as well as in microcosms experiments, is an evidence that active methanogenesis takes place *in situ* (Jones et al., 2010; Strapoc et al., 2011). Biodegradation of these compounds results in the production of metabolites such as fatty acids, and organic acids (Orem et al., 2007; Wawrik et al., 2012), which are further oxidized and fermented to methanogenic substrates. Resins and asphaltenes are detected in coal production waters indicating that these NSO-compounds are targeted by biodegradation (Orem et al., 2007). Few anaerobic degradation mechanisms for aliphatic and aromatic hydrocarbons are known. They include the condensation with fumarate and the formation of substituted succinates as catabolic intermediates (Gray et al., 2010; Strapoc et al., 2011; Wawrik et al., 2012), or the carboxylation to produce functionalized intermediates further metabolized to carbon dioxide (Head et al., 2003; Orem et al., 2010).

Microbial consortia involved in the methanogenic degradation of the organic matter in the deep subsurface have been characterized for substrates such as coal formation waters (McIntosh et al., 2008; Warwick et al., 2008; Jones et al., 2010), petroleum reservoirs production waters (Magot et al., 2000; Head et al., 2003; Roling et al., 2003; Aitken et al., 2004; Grabowski et al., 2005), and a shale-sandstone boundary (Fredrickson et al., 1997; Krumholz et al., 1997, 1999, 2002; Takai et al., 2003). The bacterial diversity is dominated by Proteobacteria, Firmicutes, Bacteroidetes, Actinobacteria, and Spirochetes depending on the substrates (Zengler et al., 1999; Grabowski et al., 2005; Green et al., 2008; Penner et al., 2010; Strapoc et al., 2011; Wawrik et al., 2012). The archaeal diversity is mostly restricted to methanogens from three families: *Methanosarcinaceae*, *Methanomicrobiaceae*, and *Methanobacteriaceae* (Zengler et al., 1999; Green et al., 2008; Penner et al., 2010; Strapoc et al., 2011). The microbial populations of oil, coal, and shale show a great diversity between substrates or between sites of the same resources. However, the putative metabolic pathways proposed for each site show a functional convergence for the anaerobic degradation of highly complex, refractory organic polymers into methane.

In a previous study, we isolated an active methanogenic consortium from an immature organic-rich shale of the Paris Basin, and showed that this consortium was able to use the OM from the shale as sole carbon and energy source (Meslé et al., 2013a). The aim of the present study is to identify which carbon fraction from the shale organic matter is used for metabolism. To address this question, we monitored methanogenesis from four different source rocks of varying maturity and partially depleted in the solvent-extractible fractions. Our results show a very strong matrix and rock source effect, but also the efficient degradation of the solvent-extractible fraction and a part of the most refractory kerogen fraction.

## Materials and Methods

### Coring and Field Sampling

Four different sedimentary rocks representing two rock types (shale and coal) and two organic matter maturities were used in this study. The shale samples belong to the Lower Jurassic black shales of the Eastern Paris Basin (France). The immature shale was sampled at ca. 7 m depth by rotation drilling (diameter of 89 mm) at Entrange (Moselle, 49°25′04.7′′N, 06°06′14.17′′E). The mature shale was collected at 1805 m depth during an exploratory drilling at Villeseneux (Marne, 48°50′31′′N, 4°08′49′′E). Low maturity coal (e.g., lignite) was sampled from an outcrop in the Languedoc region (South of France). The mature coal sample was collected at 1157 m depth during an exploratory drilling in the Lorraine region (East of France). The samples used in the experiments were taken from the center of the core and were further ground to powder using heat or ethanol sterilized material before geochemical analyses, chemical treatments, and incubation in microcosms.

### Solvent Extraction of Maltenes and Asphaltenes from the Bulk Rocks

The organic matter of the sampled rocks was gradually depleted in solvent-soluble fractions by solvent extractions performed at IFP Energies nouvelles (Rueil-Malmaison, France). All solvents were of CHROMASOLV^®^ quality (HPLC-grade) from Sigma–Aldrich (Saint-Quentin Fallavier, France). Maltenes were extracted from 100 g of grounded bulk rock (BR) sample with 200 ml of n-pentane (n-C_5_). The mix was heated (43°C) and stirred for 1 h in a glass balloon equipped with a reflux condenser, and then filtered on Büchner (0.45 μm, diameter 8 cm). The maltenes-depleted rock sample was named Residue 1 (R1). Maltenes were concentrated by n-C_5_ evaporation in a glass balloon with a rotavap, during 15 min at 43 °C and 750 mbar. Half of R1 was kept for further experimentations. The second half was submitted to dichloromethane (DCM) extraction in the same conditions to remove the asphaltenes. The maltenes- and asphaltenes-depleted rock was named Residue 2 (R2). The maltenes and asphaltenes extracted from the rock were evaporated to dryness in a rotavap at 43°C and resuspended in n-C_5_ and DCM, respectively. Residues R1 and R2 were dried overnight at 30°C to remove all traces of solvent. To quantify the soluble fractions of the OM, maltenes, and asphaltenes extracts were weighted after overnight solvent evaporation.

### Geochemical Analysis of the Rock Substrates

Geochemical characterization of the organic matter was performed by Rock-Eval 6^®^ pyrolysis (Behar et al., 2001) at IFP Energies nouvelles using 100 mg of pulverized BR, or 10 mg of R1 and R2.

### Microcosm Setup

Triplicate microcosms were constructed under anaerobic conditions as described previously (Meslé et al., 2013a). Briefly, three microcosms per rock sample were prepared in sterile 50 ml serum vials from 4 g of rock substrate (BR, R1, R2) as the only carbon source, 24 ml of organic carbon-depleted (OCD) growth medium, and inoculated with 1 ml (4%) of a 22 day-old acetate-stabilized (CP1 medium, Meslé et al., 2013a) methanogenic consortium. The OCD medium is composed of (l^-1^): 5 g NaHCO_3_, 4 g MgCl_2_ × 6H_2_O, 3.45 g MgSO_4_ × 7H_2_O, 1 g NaCl, 335 mg KCl, 250 mg NH_4_Cl, 140 mg CaCl_2_ × 2H_2_O, 140 mg K_2_HPO_4_, 2 mg Fe(NH_4_)_2_(SO_4_)_2_ × 7H_2_O, 1 mg resazurin, and 0.5 g Na_2_S. The volume is adjusted to 1:l with deionized water, sparged with N_2_/CO_2_ (80:20) gas mix and sterilized for 20 min at 121°C. The methane-producing inoculum was previously enriched from immature organic-rich shales (Lower Toarcian, Paris Basin) and subsequently isolated from its rock matrix by at least two transfers on CP1 medium. Its ability to produce methane from the immature organic-rich shale as the only carbon source was confirmed before its utilization as inoculum in this experiment (Meslé et al., unpublished results). Prior to inoculation, the consortium was washed twice in OCD medium to remove traces of organic carbon from the culture medium. Control experiments included microcosms set up with acetate and rock sample but no inoculum to evaluate the presence of live methanogenic communities (Indigenous methanogen control), acetate but no rock as carbon source (inoculum positive controls), no carbon source (inoculum negative controls, e.g., no acetate and no rock sample) and heat sterilized (autoclaved 20 min at 121°C in a sealed serum bottle under N_2_ atmosphere) BR (Sterilized BR). Vials were sealed with a rubber cap and a metal ring, and anoxic conditions in the microcosms were obtained by atmosphere substitution with N_2_/CO_2_ (80:20) and the addition of Na_2_S (0.5 g l^-1^). The microcosms were incubated at a constant temperature of 25°C in the dark without shaking for three and a half months.

### Monitoring of Methane Production

Methane production was monitored by gas chromatography, by direct injection of 10 μl of headspace gas into a 7820A gas chromatograph (Agilent Technologies, Massy, France) equipped with a thermal conductivity detector (TCD) and a flame ionization detector (FID). Methane concentrations are obtained against a calibration curve prepared with pure CH_4_ (Air Liquide, France) and expressed in μmoles/g rock (dry weight). The SD was calculated based on GC-FID measurements on triplicate cultures. Methane production was monitored at 30, 43, 72, and 114 days of incubation.

### gDNA Extraction

Molecular analyses were performed on microcosms containing the immature shale samples (sterilized BR, unsterilized BR, R1, and R2) and on microcosms corresponding to the positive controls, at day 155 of incubation. The 22-day-old consortium used as inoculum in this experiment was also analyzed as a reference. The triplicate microcosms were homogenized by inversion, and 2 ml of culture were sampled using a 2 ml syringe. Samples were centrifuged for 5 min at 13,000 rpm, and the supernatant discarded. The pellet was used for gDNA extraction with the Ultraclean^®^ Soil DNA Isolation Kit (MO BIO Laboratories, Carlsbad, CA, USA). gDNA yield was quantified using the Quantit^TM^ dsDNA HS Assay Kit and the Qubit^TM^ fluorometer (Invitrogen, Eugene, OR, USA). gDNA quality was evaluated by PCR of the bacterial and archaeal 16S rRNA genes.

### Phylogenomic Studies

Amplicon libraries of 16S rRNA genes were prepared as detailed by Meslé et al. (2013a) on triplicate gDNAs extracted from all microcosms, using the primer pair 1073F/787R for Bacteria and the primer pair 571F/a910R for Archaea (both described in Meslé et al., 2013a). The same tag combination was used for the triplicate microcosms, and equimolar amounts of the purified amplicons were pooled before sequencing, in order to obtain an average microbial composition for each condition as shown by Uroz et al. (2010). Four hundred fifty-four sequencing was performed on a genome sequencer (GS) Junior Titanium Series (Roche Applied Science, Meylan, France) at Biofidal (Vaulx-en-Velin, France), according to the manufacturer’s instructions for amplicons sequencing. Taxonomic analyses were performed as indicated by Meslé et al. (2013a). Sequence data have been deposited in the SRA archive at NCBI.

## Results

### Geochemistry of the Bulk Rocks and Their Extraction Residues

**FIGURE 1:**
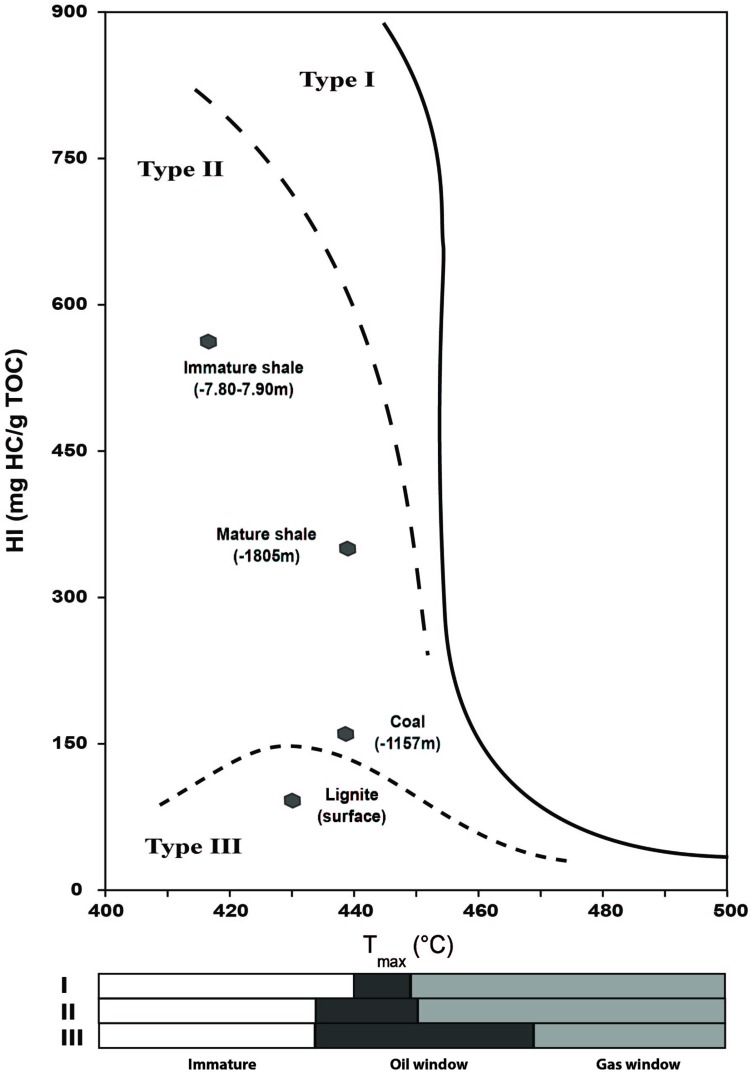
**HI and T_max_ crossed-diagram of the bulk rock (BR) samples analyzed by Rock-Eval 6 pyrolysis**.

Rock-Eval results for the four rock samples are given in **Table 1**. Values obtained from the two shales are consistent with the expected profile of Type II kerogen-rich rocks (Tissot et al., 1974; Durand, 2003), as observed on the crossed diagram of HI and Tmax values (**Figure 1**) proposed by Espitalié et al. (1977). These two samples belong to the same source rock, but have undegone different evolutions in terms of temperature and pressure in their burial history, which is reflected by their geochemical properties. The shallower shale presents Tmax and HI values value of 418°C and 575 mg HC/g total organic carbon (TOC), respectively, (**Figure 1**; **Table 1A**) indicating an immature source rock. The second shale, sampled at a depth of 1800 m, has a higher Tmax value of 440°C and a lower HI value of 366 mg HC/g TOC, indicating a range of maturity within the oil window for Type-II kerogen. The TOC value of the immature shale is 7.98 wt% vs. 2.11 wt% for the mature shale (**Table 1A**).

**Table 1:** Main geochemical parameters obtained by Rock-Eval 6 pyrolysis on the four bulk rocks **(A)** and their residues after solvent extractions, **(B)** Residue 1: after n-C_5_ extraction, **(C)** Residue 2: after n-C_5_ and dichloromethane (DCM) extraction, **(D)** Quantifications of the soluble fractions extracted from the bulk rocks (BRs).

(A)							

**Bulk Rock**	**S1 (mg/g TOC)**	**S2 (mg/g rock)**	**T_max_ (°C)**	**Total organic carbon (TOC) (wt%)**	**HI (mg HC/g TOC)**	**OI (mg C02/g TOC)**	**MINC (wt%)**
Immature shale	6.76	42.03	418	7.98	575	25	3.19
Mature shale	32.17	7.52	440	2.11	366	20	0.49
Lignite	0.87	40.84	431	46.60	104	80	3.99
Coal	2.87	72.78	439	43.85	167	4	1.02

**(B)**							

**Residue 1**							
Immature shale	2.10	37.89	415	9.54	398	25	
Mature shale	5.28	5.04	438	2.75	184	31	
Lignite	0.84	33.05	429	38.12	87	79	
Coal	3.39	52.87	435	31.46	168	8	

**(C)**							

**Residue 2**							
Immature shale	0.75	35.22	414	9.37	376	27	
Mature shale	3.06	5.11	438	2.615	195	40	
Lignite	0.74	33.91	432	38.73	88	78	
Coal	1.62	47.60	436	31.278	152	7	

**(D)**							
	**n-C_5_**	**DCM**					
**Extracts**	**Maltenes (mg/g TOC)**	**Asphaltenes (mg/g TOC)**					

Immature shale	9.10	38.28					
Mature shale	28.96	30.12				
Lignite	0.31	4.50				
Coal	0.58	6.29				

The free hydrocarbons, represented by the S1 value, were similarly normalized to the total organic carbon content (TOC values) of the BR. Their higher proportion in the mature shale, i.e., 32.17 mg/g TOC vs. 6.76 mg/g TOC in the immature shale, is also an illustration of the thermal cracking undergone by the deep shale sample (**Table 1A**). The solvent-extractible fraction (represented in part by the S1 value), were also normalized to the TOC values of the BR (**Table 1D**). Their respective concentrations also illustrate the thermal evolution of the two shale samples. The OM of the mature shale presents a higher proportion of maltenes than the immature shale, ca. 29 mg/g TOC vs. 9 mg/g TOC, respectively. This result is consistent with a thermal cracking of the kerogen, which releases light molecules such as saturates, aromatics, and polar compounds (Lewan and Ruble, 2002; Durand, 2003; Vandenbroucke, 2003; Behar et al., 2008). Thermal cracking also affects asphaltenes, which explains their lower proportion in the mature shale (**Table 1D**) compared to the immature shale. The organic matter in lignite and coal samples shows the expected profile for Type III kerogen although the coal presents a high HI of 142 mg HC/g TOC, twice higher than the HI of the lignite, i.e., 77 mg HC/g TOC (**Figure 1**; **Table 1A**). The crossed diagram of HI and Tmax values reveals a coal at the beginning of the oil window for a Type III organic matter, with a Tmax of 437°C. The lignite is very close to the oil window but is still immature, with a Tmax of 429°C. Compared to the shales, their TOC is much higher, 39 wt% for the lignite and 21 wt% for the coal. Both present a similar oil potential S2 around 29 mg/g rock, and a low amount of free hydrocarbons, ca. 0.77 mg/g TOC and 2 mg/g TOC for lignite and coal, respectively. The concentrations of maltenes and asphaltenes in Type III organic matter are very low compared to those observed in the shale (**Table 1A**), and asphaltenes largely exceed maltenes (**Table 1D**), which is fully consistent with the kinetic scheme proposed by Behar et al. (2008).

Rock-Eval pyrolysis data obtained on Residue 1 (**Table 1B**) and Residue 2 (**Table 1C**) revealed a progressive decrease of the HI and S1 values of each rock. This trend illustrates the efficiency of the solvent extraction to remove the soluble fractions from the rock. The slight increase of the TOC values of the two shales from BR to R2 may be linked to the presence of pyrite in these source-rocks which were deposited in a marine environment (Vandenbroucke and Largeau, 2007) and/or to the removal of elemental sulfur (S°) during solvent extraction. Theoretically, values for the Residue 2 (bitumen-depleted rock) reflect the quality of the kerogen, which is the only remaining source of organic carbon at this stage. The Residue 2 of the immature shale presented the highest HI value (376 mg HC/g TOC). This result is not surprising, since an immature Type II kerogen usually contains more aliphatic chains than a mature Type II kerogen or a Type III kerogen (Vandenbroucke, 2003). The TOC is another key parameter quantifying the kerogen in the residues R2 (**Table 1C**). It shows that lignite and coal contain a higher proportion of kerogen in their organic matter (ca. 31–38 wt%) compared to the shales (ca. 2–9 wt%).

### Methane Production From the Rock Substrates in Microcosm

**FIGURE 2:**
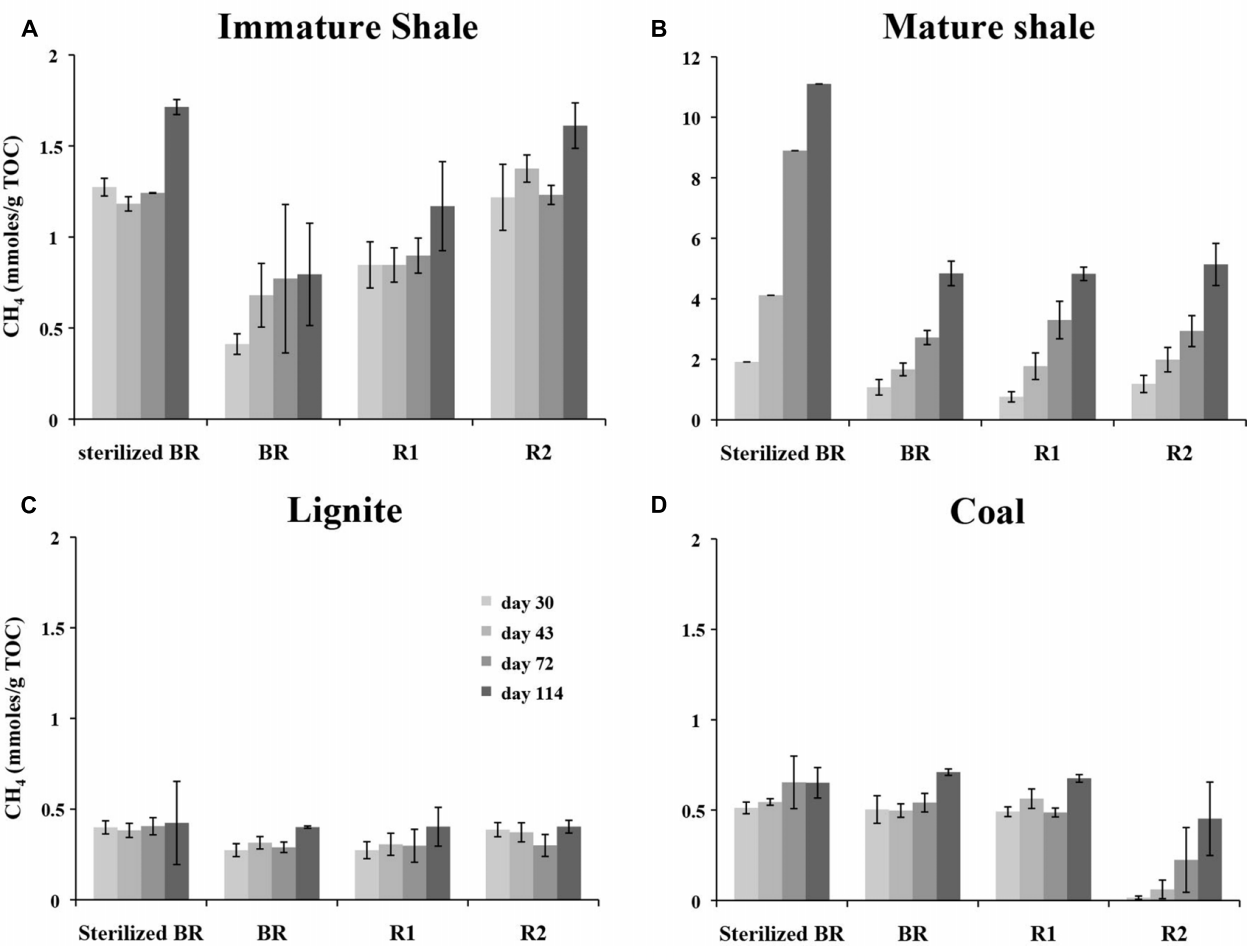
**Methane accumulation in the microcosms containing the different substrates prepared from the four sedimentary rocks sampled. (A)** Immature Shale; **(B)** Mature Shale; **(C)** Lignite; **(D)** Coal. The SD was calculated based on GC-FID measurements on triplicate cultures. Methane production was normalized to the total organic carbon (TOC) value of each susbtrate in order to highlight the possible divergent efficiencies in the utilization of the organic matter between the rock substrates. BR, Bulk Rock; R1, Residue 1; R2, Residue 2.

Methane production from microcosms was monitored at 30, 43, 72, and 112 days of incubation (as described in Meslé et al., 2013a). Methane production and methanogens remained below the detection limits in the three Indigenous methanogen community controls (Coal, Lignite, and Mature Shale), indicating that if live methanogenic communities were present, their number were extremely low, and they could not be cultivated in CP medium. In contrast, significant methane production was detected in all inoculated microcosms containing rock samples (**Figure 2**). Up to 800 μmoles of methane were produced in the positive, acetate-containing controls, whereas no methane was detected in negative control microcosms setup without carbon source. The largest methane production was observed in microcosms prepared with the mature shale, regardless of solvent extraction (BR, R1, R2). Microcosms containing the R2 yielded ca. 5.7 mmoles/g TOC at day 114 (**Figure 2B**), but methane accumulation was much larger and faster from the sterilized BR, reaching ca. 10.9 mmoles/g TOC at day 114. Methane accumulation was lower in the microcosms prepared with the immature shale substrates (**Figure 2A**). In contrast to the mature shale, we observed an increase in CH4 accumulation from the unsterilized BR (ca. 0.8 mmoles/g TOC at day 114) to the Residue 2 (ca. 1.8 mmoles/g TOC) of the immature shale. Although not as strong, we also observed higher methane accumulation in the microcosms containing the heat sterilized BR sample (ca. 1.7 mmoles/g TOC). Comparatively, methane production from lignite and coal was much lower and did not exceed 0.7 mmoles/g TOC (**Figures 2C,D**). The average methane concentration at day 114 is equivalent from all the different lignite-based substrates, ca. 0.4 mmoles CH_4_/g TOC. A different pattern of methane accumulation was observed for coal. Similar methane accumulations were observed from the sterilized and unsterilized BR, as well as from the Residue 1, ca. 0.6–0.7 mmoles/g TOC at day 114 (**Figure 2D**). However, methane production from Residue 2 is slower and lesser: a significant amount of methane could only be detected after 43 days of incubation, and methane concentration reached ca. 0.45 mmoles/g TOC at day 114.

### Sequences Analysis

**FIGURE 3:**
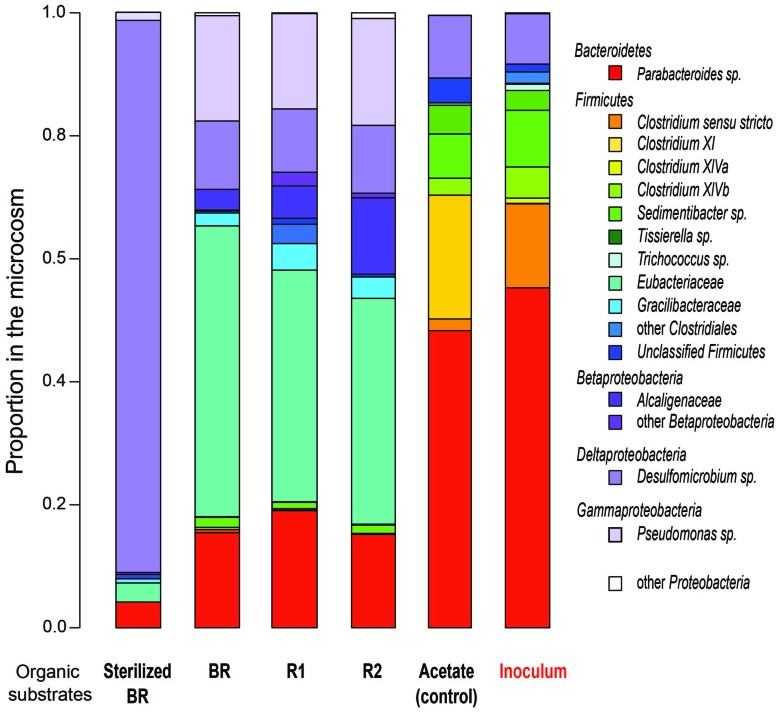
**Comparative bacterial diversity after 155 days of incubation in the different microcosms containing substrates prepared from the immature shale sample, and in the acetate-grown consortia (control and inoculum).** BR, Bulk Rock; R1, Residue 1; R2, Residue 2.

The influence of the carbon source on microbial population structure in microcosm was evaluated by 454-pyrosequencing of the 16S rRNA bacterial and archaeal gene libraries from microcosms constructed with the immature shale and residues. More than 9500 bacterial 16S partial sequences were obtained and defined 387 OTUs for the six conditions sampled, although only three phyla were identified: Bacteroidetes, Firmicutes, and Proteobacteria (**Table 2**). A large majority of the OTUs defined was present in the different microcosms, and sequences from the 10 most abundant and best-shared OTUs represented 50 to 84% of the total number of sequences (**Table 3**). However, bacterial diversity shows a shift in terms of taxonomy between the consortium maintained on acetate and the consortia grown on the rock substrates (**Tables 2–4**). Initially, the inoculum presents a very low bacterial diversity, since the 10 most abundant OTUs represent over 92% of the total Bacteria (**Table 4**), as opposed to the acetate control (ca. 66%) or the unsterilized rock-containing microcosms (ca. 60 to 72%). The low Chao and Shannon indexes of this sample (**Table 2**) confirmed this observation. When maintained on acetate (positive control), the bacterial diversity of the inoculum does not evolve strikingly. Although more OTUs were defined in the control compared to the inoculum (51 vs. 40 OTUs, respectively), 9 out of the 10 most abundant and best-shared OTUs are maintained (**Table 3**) and the diversity profiles are very similar (**Figure 3**), with Bacteroidetes representing half of the total Bacteria present (**Table 2**). *Parabacteroides* is the only genus of this phylum detected in all our microcosms (**Table 5**). In the positive control and inoculum, this genus is represented by two major OTUs accounting for ca. 36 and 54% of the total bacterial diversity, respectively.

**Table 2:** Bacterial diversity at the phylum level in the microcosms prepared from the immature shale (7 m depth).

		Organic substrates						
	OTU	Sterilized BR	BR	R1	R2	Acetate (control)	Inoculum
Bacteroidetes	45	4.18	15.46	19.02	15.19	48.31	55.32
Firmicutes	204	3.76	52.46	47.6	42.33	41.53	36.35
Proteobacteria	138	92.06	32.09	33.38	42.48	10.17	8.33
Chao		985	943	1048	1842	964	131
Shannon		2.81	3.99	4.41	4.30	4.29	2.33

*Values reported are percentages of sequences representing each phylum for the triplicate microcosms. OTU, number of OTUs defining the phylum. BR, Bulk Rock; R1, Residue 1; R2, Residue 2. Chao, normalized diversity index of Chao. Shannon, normalized diversity index of Shannon.*

**Table 3:** Proportion of the 10 most abundant and best-shared OTUs from the microcosms prepared with the immature shale-derived substrates.

			Organic substrates					
Phylum	Genus	OTU #	Sterilized BR	BR	R1	R2	Acetate (control)	Inoculum
Bacteroidetes	*Parabacteroides*	1563	0.49	3.74	3.14	3.17	14.62	16.61
	*Parabacteroides*	1568	2.65	7.01	8.31	7.71	20.97	37.67
Firmicutes	Unidentified Eubacteriaceae	24	0.63	10.49	11.5	9.07	–	–
	Unidentified Eubacteriaceae	1550	1.39	14.74	9.88	12.02	–	–
	*Clostridium sensu stricto*	1604	–	0.26	0.09	–	1.06	13.58
	Unidentified Eubacteriaceae	1610	1.05	14.12	8.77	10.05	–	–
	*Sedimentibacter*	1629	–	0.31	0.23	0.23	3.39	7.57
Proteobacteria	*Desulfomicrobium*	5	18.12	1.13	1.94	1.97	2.12	–
	*Desulfomicrobium*	1556	57.77	8.65	6.93	7.71	7.63	8.19
	*Pseudomonas*	1559	0.84	5.94	5.17	6.5	–	–
			**82.94**	**66.39**	**55.96**	**58.43**	**49.79**	**83.62**

**Table 4:** Proportion of the 10 most abundant OTUs for each sample.

Organic substrates	

	**Sterilized BR**		**BR**		**R1**		**R2**		**Acetate (control)**		**Inoculum**
57.77	*Desulfomicrobium*	14.74	Unidentified Eubacteriaceae	11.50	Unidentified Eubacteriaceae	12.02	Unidentified Eubacteriaceae	20.97	*Parabacteroides*	37.67	*Parabacteroides*
18.12	*Desulfomicrobium*	14.12	Unidentified Eubacteriaceae	9.88	Unidentified Eubacteriaceae	10.05	Unidentified Eubacteriaceae	14.62	*Parabacteroides*	16.61	*Parabacteroides*
3.00	*Desulfomicrobium*	10.49	Unidentified Eubacteriaceae	8.77	Unidentified Eubacteriaceae	9.07	Unidentified Eubacteriaceae	7.63	*Desulfomicrobium*	13.58	*Clostridium sensu stricto*
2.65	*Parabacteroides*	8.65	*Desulfomicrobium*	8.31	*Parabacteroides*	7.71	*Desulfomicrobium*	5.93	*Clostridium XI*	8.19	*Desulfomicrobium*
1.39	Unidentified Eubacteriaceae	7.01	*Parabacteroides*	6.93	*Desulfomicrobium*	7.71	*Parabacteroides*	4.87	*Parabacteroides*	7.57	*Sedimentibacter*
1.05	Unidentified Eubacteriaceae	5.94	*Pseudomonas*	5.17	*Pseudomonas*	6.50	*Pseudomonas*	3.39	*Sedimentibacter*	4.87	*Clostridium XlVb*
0.91	*Desulfomicrobium*	3.74	*Parabacteroides*	3.14	*Parabacteroides*	4.76	Unidentified Alcaligenaceae	2.54	*Parabacteroides*	1.14	*Tissierella*
0.84	*Pseudomonas*	3.02	*Pseudomonas*	2.91	*Pseudomonas*	3.78	*Pseudomonas*	2.33	*Sedimentibacter*	1.09	other Clostridiales
0.77	*Desulfomicrobium*	3.02	*Pseudomonas*	1.99	*Parabacteroides*	3.17	*Parabacteroides*	2.12	*Clostridium XI*	1.04	*Trichococcus*
0.70	*Desulfomicrobium*	1.74	Unidentified Alcaligenaceae	1.94	*Desulfomicrobium*	2.57	Unidentified Alcaligenaceae	2.12	*Desulfomicrobium*	0.76	*Clostridium XlVa*
87.18		72.47		60.53		67.35		66.53		92.52	

**Table 5:** Taxonomic affiliation of the OTUs defined for the different substrates prepared from the immature shale sample, and for the acetate-grown consortia (control and inoculum).

			Organic substrates									
Phylum	Class	Genus	Sterilized BR		BR		R1		R2		Acetate (control)		Inoculum	
			OTU	%	OTU	%	OTU	%	OTU	%	OTU	%	OTU	%
Bacteroidetes		*Parabacteroides sp.*	7	**4.18**	20	**15.46**	28	**19.02**	12	**15.19**	11	**48.31**	6	**55.27**
Firmicutes		*Clostridium sensu stricto*	0	–	3	**0.51**	2	**0.18**	0	–	3	**1.91**	2	**13.66**
		*Clostridium XI*	0	–	0	–	2	**0.23**	1	**0.15**	17	**20.13**	1	**0.09**
		*Clostridium XlVa*	0	–	0	–	0	**-**	0	–	0	–	2	**0.85**
		*Clostridium XlVb*	0	–	2	**0.36**	2	**0.182**	0	–	2	**2.75**	2	**5.06**
		*Sedimentibacter sp.*	0	–	10	**1.64**	5	**0.78**	5	**1.36**	5	**7.2**	6	**9.22**
		*Tissierella sp.*	0	–	1	**0.1**	1	**0.09**	1	**0.15**	4	**4.66**	6	**3.22**
		*Trichococcus sp.*	0	–	0	–	0	–	0	–	1	**0.42**	1	**1.04**
		Unidentified Eubacteriaceae	4	**3.07**	43	**47.29**	45	**37.67**	21	**36.73**	0	–	0	–
		Unidentified Gracilibacteraceae	3	**0.7**	7	**2.1**	7	**4.34**	6	**3.48**	0	–	1	**0.14**
		Other Clostridiales	0	–	2	**0.31**	3	**3.14**	0	–	0	–	5	**1.84**
		Unclassified Firmicutes	0	–	1	**0.15**	5	**0.97**	2	**0.45**	5	**4.03**	6	**1.28**
Proteobacteria	Betaproteobacteria	Unidentified Alcaligenaceae	2	**0.7**	5	**3.38**	7	**5.26**	14	**12.4**	0	–	0	–
		Other Betaproteobacteria	1	**0.35**	0	–	1	**2.22**	2	**0.76**	0	–	0	–
	Deltaproteobacteria	*Desulfomicrobium sp.*	46	**89.76**	9	**11.11**	14	**10.3**	8	**11.04**	3	**10.17**	1	**8.18**
	Gammaproteobacteria	*Pseudomonas sp.*	4	**1.25**	20	**17.14**	24	**15.51**	14	**17.38**	0	–	0	–
		Other Proteobacteria	0	–	3	**0.46**	1	**0.09**	5	**0.91**	0	–	1	**0.14**

*OTU, number of separate OTUs at a given taxonomic level. %, indicates the percentage of the total sequences composing a group of OTUs.*

A second diversity profile was observed in all the unsterilized rock samples (BR, R1, and R2). For a number of sequences normalized to the sample least covered, we observed an increase in the richness (Chao index) in the microcosm containing the Residue 2 (**Table 2**). Bacterial diversity does not show a strong reduction trend in the BR, R1, and R2 microcosms since the Shannon index remains stable (**Table 2**). However, a few variations in the OTU numbers and taxonomy were observed. The proportion of Firmicutes decreases from BR to R2 (52 to 42%) but is higher than in the acetate-grown consortia. This decrease is correlated with a 10%-increase in the proportion of Proteobacteria from BR to R2 (ca. 32 to 42%), largely above the proportion detected on acetate (ca. 10%). The phylum Bacteroidetes represents less than 20% of the bacterial diversity in the microcosms containing the unsterilized rock substrates, a much lower proportion than in the consortium grown on acetate (**Table 2**).

Microcosms containing the sterilized BR present a very distinct diversity pattern composed of ca. 92% of Proteobacteria (**Table 2**) represented exclusively by the genus *Desulfomicrobium* (**Table 5**; **Figure 3**), with three major OTUs out of 46 accounting for ca. 79% of the total bacterial diversity (**Table 4**). When grown on the sterilized BR, the consortium thus exhibits a lower bacterial diversity than the consortium grown on unsterilized rock substrates (BR, R1, and R2), as confirmed by the lower Chao and Shannon indexes (**Table 2**). *Desulfomicrobium* was detected in all the other microcosms with an average proportion of 10% (**Table 5**). Amongst the 204 OTUs composing the Firmicutes, sequences related to the genera *Clostridium sensu stricto*, *Clostridium* XlV a and b, *Sedimentibacter*, *Trichococcus*, and *Tissierella* were identified in the acetate-grown consortia (inoculum and control), whereas several yet unidentified *Eubacteriaceae* and *Gracillibacteraceae* are the most abundant *Firmicutes* in the consortia grown on the unsterilized rock substrates (**Table 4**; **Figure 3**). Amongst Proteobacteria, several unidentified *Alcaligenaceae* were restricted to the unsterilized rock samples (**Table 5**). *Pseudomonas* represents up to 17% of the bacterial diversity in the consortia grown on BR, R1, and R2 (four OTUs, one major), but is not seen in the acetate-grown consortia (**Table 4**; **Figure 3**). The dominant OTU of the genus *Parabacteroides* is conserved between the different carbon substrates (**Table 4**).

The consortium used as inoculum in the different microcosms presented very low archaeal diversity, since only species of the genus *Methanosarcina* were present. Thus, it was not surprising to identify only *Methanosarcina* in the microcosms inoculated with this consortium. Pyrosequencing data revealed that the major OTU present in the inoculum was maintained on the different organic substrates, including acetate. This OTU accounted for 82.7, 78.74, 73.13, 58.84, and 74.78% of the archaeal diversity in the microcosms prepared from unsterilized BR, R1, R2, acetate, and sterilized BR, respectively. The rest of the archaeal diversity in these microcosms is represented, respectively, by 16, 26, 15, 12, and 16 others OTUs belonging to the genus *Methanosarcina*.

## Discussion

This study aimed at defining the metabolic profile of active methanogenic consortia living in organic-rich shales from the Paris Basin. A stabilized methanogenic consortium originally isolated from the Paper Shales formation of the Eastern border of the Paris Basin was grown in presence of four different rocks, presenting two different maturities, and two different kerogen types. Our results confirm and extend the preferential growth substrates of active methanogenic consortia from bitumen to the more complex kerogen. This study shows that the organic matter from the four sedimentary rocks studied was degraded into methane in less than 30 days by the active microbial consortium isolated, regardless of the compositional differences of these rocks. However, different methane production patterns were observed. Shale substrates yielded higher methane than coal and lignite. Furthermore, mature rocks yielded more methane than their immature source-rocks. These observations are consistent with previous results: shales contain the highest proportion of maltenes and asphaltenes in their organic matter (**Table 1D**), molecules that are efficiently biodegraded by anaerobic microorganisms (Walker et al., 1976; Magot et al., 2000; Formolo et al., 2008). Likewise, the highest methane accumulation was assessed on the mature shale, which contains the highest proportion of maltenes that have been produced by thermal cracking during burial (Tissot et al., 1974; Tissot, 1977; Deming and Baross, 1993). It is interesting to note the ability of our consortium isolated from immature, shallow organic-rich shales, to degrade the organic matter from a mature shale, but also the organic matter of mature and immature Type III rocks. These results show that our methanogenic consortium is able to grow and produce methane from organic substrates corresponding to the total and unperturbed organic matter from the BR, i.e., maltenes, asphaltenes, and kerogen, as well as from rocks progressively depleted in maltenes (R1) and asphaltenes (R2). This clearly indicates that the consortium was able to degrade not only the soluble fractions of the organic matter present in the rocks, but also at least a part of the organic fraction insoluble in solvents, i.e., kerogen. In our experiment, we observed a residual fraction of soluble organic molecules as shown by the non-null S1 values of the R2 residues (ca. 0.76 to 3.06 mg/g TOC, **Table 1C**). However, no correlation could be established between methane quantities produced in the microcosms set up with R2 residues (**Figure 2**) and the amount of these residual organics (S1, **Table 1C**). The majority of methane produced under these conditions must come from the degradation of the kerogen itself.

Methane accumulation from the residue R2 of coal is low and much slower than it is from the R2 of the other three rock types. This is not unexpected. Type III kerogen as contained in coal is composed for a large part of ligneous debris with an aromatic structure, together with aliphatic protective coatings from the rigid skeleton of higher plants (Vandenbroucke, 2003; Vandenbroucke and Largeau, 2007). The thermal cracking of this kerogen releases C6–C35 n-alkanes (Behar and Vandenbroucke, 1988), and the remaining lignin, which is known to be resistant to both thermal and biological degradation, contributes to the high aromaticity of the mature Type III kerogen (Behar et al., 1986; Vandenbroucke, 2003; Vandenbroucke and Largeau, 2007). Thus, a mature coal located in the oil window for Type III kerogen such as our coal sample should have a predominantly aromatic structure and be more recalcitrant to biodegradation, resulting in the low methane yield observed (**Figure 2D**). The faster rate of methane production in the immature kerogen from the lignite, which chemical structure is less complex than that of coal, seems to support this view (**Figure 2C**). In comparison to coal and lignite, the chemical structure of Type II kerogen contains more aliphatic chains and naphtenes (Behar et al., 1986; Vandenbroucke, 2003). The shales were expected, and were found, to yield higher methane production than coal and lignite. However, opposite to our observation in coal and lignite, methane yields were threefold higher for the R2 of the mature shale than for the R2 of the immature shale (ca. 5 against 1.6 mmoles CH_4_/g TOC, respectively, **Figures 2A,B**). According to the chemical structure of the immature Type II kerogen, richer in aliphatic and naphtenic carbon than the more mature Type II kerogen, we expected a higher methane accumulation from the Residue 2 of the immature shale. Behar and Vandenbroucke (1988) proposed that hydrocarbons are trapped inside the kerogen matrix in type II kerogens. The higher S1 value in the R2 of the mature shale shows that some small organics were not extracted during our experiment. However, the total quantity available is insufficient to explain the difference in methane production between the mature and immature shales. A deeper geochemical analysis, especially one targeting the chemical structure of the kerogen matrix, would be necessary to evaluate the relative proportion of methyl groups, alkyl chains, and heteroatoms that are preferential targets of microbial degradation (Strapoc et al., 2011).

The very similar methane yields obtained from the differentially depleted residues (i.e., BR, R1, and R2), a profile observed for the two types of source rocks used in our study (i.e., shale and coal), is surprising. Maltenes, the light compounds of the organic matter have been shown to be the preferential microbial substrates and to be the first molecules effectively degraded during the biodegradation of oil (Head et al., 2003). Thus, a better methane yield was expected from fractions containing maltenes (BR) than from the R1 and R2 in which maltenes were removed by solvent extraction. On the contrary, under our experimental conditions we observed better methane yields for R1 and R2. This demonstrates that our consortium can efficiently degrade asphaltenes and kerogen. It may also reflect a low accessibility to the organic resource in the BR. Indeed, bitumen has a colloidal structure, with an asphaltenic core coated by resins, and aggregated in an oily dispersion medium constituted by saturates and aromatics (Merdrignac and Espinat, 2007; Lesueur, 2009). Asphaltenes are insoluble in low carbon number n-alkanes, such as n-heptane, or n-hexane, while maltenes are soluble in such solvents (Tissot and Welte, 1984). Our results indicate that the consortium might encounter difficulties to degrade all together the organic fractions in the BR. One can suppose that the consortium cannot access the high molecular weight fractions in the untreated BR, resulting in a lower or equivalent methane yield from the BR compared to the R1 and R2. Whether the kerogen is coated by the bitumen fractions or mixed inside the mineral matrix remains unclear. Hypothetical structural models of kerogen have been proposed since the late 1960s, as the representation of the building blocks of kerogen structure by Burlingame and co-workers (Burlingame and Simoneit, 1969), or the first detailed molecular representation of kerogen by Yen (1976). This latter suggests that bitumen molecules may be entrapped within the kerogen matrix, which was later confirmed by Behar and Vandenbroucke (1988). The successive chemical treatments with the organic solvents have likely modified the chemical structure of the OM in the rock. The removal of the bitumen fractions from the BRs could have extended the contact zone between water and the kerogen, increasing the accessibility to the microbial consortium of heavy OM fractions remaining in the residues R1 and R2. Increased accessibility could explain why (1) there is no decrease in methane yield with the depletion of the most soluble and degradable fractions, and (2) a production of methane occurs from fractions that only contain kerogen as carbon source (Lesueur, 2009; Gaspar et al., 2012).

Pyrosequencing data revealed a shift in bacterial diversity between the acetate-stabilized consortia (inoculum and positive control) and the consortia grown on the immature shale substrates (i.e., unsterilized BR, R1, and R2). The bacterial diversity of acetate-grown consortia is mainly represented by two genera, *Clostridium* and *Parabacteroides*, which are present in lower proportion in consortia grown on the immature shale substrates (**Table 5**). *Parabacteroides* is an obligate fermenter capable of degrading organic polymers such as cellobiose (Sakamoto and Benno, 2006; Sakamoto et al., 2007). Species of the genus *Clostridium* are known as fermenting organisms, accepting a wide range of substrates such as various carbohydrates (Schnurer et al., 1996), cellulose (Ng et al., 1977), or amino acids (Hoogerheide and Kocholaty, 1938). They can oxidize anaerobically fatty acids, as well as propionate and acetate, to hydrogen and carbon dioxide in association with hydrogen-consuming organisms (Stieb and Schink, 1985; Schnurer et al., 1996). The fermentation end-products of *Clostridium* species are various: acetate, propionate, isobutyrate, butyrate, iso-valerate, and valerate (Borsodi et al., 2003). Other putative acetate-producing fermenters are detected at significant levels inside the microcosms containing acetate (e.g., *Tisierella* and *Sedimentibacter*), which are capable of fermenting fatty acids and other hydrocarbons to acetate (Borsodi et al., 2003). The metabolism of these organisms is in good agreement with the composition of the CP1 medium, which contains peptides and yeast extract in addition to acetate as carbon sources. The shale-derived inoculum thus contains a complete methanogenic consortium, composed of fermentative and acetogenic bacteria (e.g., syntrophs) represented mostly by diverse *Firmicutes*, and a main genus of *Bacteroidetes*, i.e., Parabacteroides; associated with methanogenic archaea. When grown in presence of shale or shale-derived samples (unsterilized BR, R1, and R2), the bacterial populations exhibited a majority of *Eubacteriaceae* species, which were not detected in the inoculum and control microcosms. This family is composed of fermenting and acetogenic bacteria able to degrade carbohydrates, amino acids, cellulose, and pyruvate into small molecular organic acids such as acetate (Guo et al., 2012). Other sugar-fermenting bacteria, belonging to the family *Alcaligenaceae* and *Gracilibacteraceae*, were also detected (Deley et al., 1986; Coenye et al., 2005; Lee et al., 2006). Finally, the genus *Pseudomonas* represented ca. 16% of the bacterial diversity in these microcosms. Species of this genus have been previously involved in the degradation of hydrocarbons in oil reservoirs (May and Neihof, 1982; Widdel and Rabus, 2001; Emtiazi et al., 2005; Grabowski et al., 2005). *Pseudomonas* may come from the immature shale substrate added in these microcosms, since the BR was grounded in aerobic conditions and has undergone a solvent extraction, two steps that certainly eliminated the indigenous microorganisms. The bacterial diversity is similar in all shale-containing microcosms as shown by the similar Shannon indexes. These results suggest that these Bacteria are able to metabolize the different fractions of the organic matter from the shale. This observation could support the hypothesis of an increased accessibility of the organic fractions allowing the same consortium to utilize the different fractions of the OM.

The shift in bacterial diversity between the acetate-grown consortia and the shale-grown consortia seems to confirm that the bacterial diversity is substrate-related. When grown on the shale substrates (unsterilized BR, R1, and R2), the inoculum has evolved to a hydrocarbon-degrading methanogenic consortium compared to the control grown on acetate. Solvent extraction on the immature shale seems to be correlated with a decrease in the proportion of *Firmicutes* replaced by Proteobacteria, without apparent loss of diversity (**Table 2**). Whether this microbial evolution is linked to the slight increase in methane production from BR to R2 remains unknown. Most of the microorganisms identified in the acetate- and shale-grown consortia have been previously detected in coalbed methane formations of diverse sedimentary basins (Strapoc et al., 2008; Jones et al., 2010; Dawson et al., 2012; Wawrik et al., 2012), as well as in biodegraded oil reservoirs (Magot et al., 2000; Grabowski et al., 2005). The ability of these fermenting and homoacetogenic bacteria to hydrolyze macromolecules was evidenced (Dawson et al., 2012; Zakrzewski et al., 2012). Thus, in spite of an evolving bacterial diversity, the consortium maintains its methanogenic ability whatever the available organic substrate.

This observation is supported by the pyrosenquencing data obtained from the consortium grown on the sterilized BR. The genus *Desulfomicrobium*, which contains sulfate-reducing bacteria (SRB), accounted for almost 90% of the total bacterial diversity on this substrate. Type II organic matter originates from marine sediments and often contains pyrite (FeS2) resulting from the alteration of organic molecules by SRB (Vandenbroucke and Largeau, 2007). Disulfide bonds, present in pyrite, are very sensitive to heat thus the sterilization of the rock at 121°C for 20 min might have dissociated the bonds and oxidized the OM. The subsequent release of oxidized sulfur could explain the dominance of SRB on the sterilized immature shale. The capacity of SRB belonging to the phylum Proteobacteria to degrade saturated and aromatic hydrocarbon has been previously evidenced (Widdel and Rabus, 2001), and SRB are known to utilize oil components directly (Aeckersberg et al., 1991; Rueter et al., 1994; Spormann and Widdel, 2000; Kniemeyer et al., 2007; Musat et al., 2009). Their potential association with the *Methanosarcina* species could explain the efficient methanization of the sterilized BR, but these assumptions need to be further investigated by 454 sequencing on other sterilized shale susbtrates. The break-up of weak chemical bonds in the complex molecular structure of kerogen by heat sterilization may also explain the overall higher methane yields obtained from the different sterilized BRs.

From this study and our previous investigations on the immature organic-rich shales of the Paris Basin, it is clear that these substrates represent a good compromise between organic richness and methanogenic potential. Although the mature shale yielded higher methane accumulation, this substrate is unlikely to contain active indigenous microbial consortia *in situ* regarding its localization at 1800 m depth in the beginning of the oil window, where the temperature is above 80°C and unfavorable to microbial growth and biodegradation activity (Magot et al., 2000). Immature shales of the Eastern Paris Basin naturally contain indigenous microbial consortia composed of syntrophic bacteria associated with methanogenic archaea, able to mineralize efficiently the different molecular fractions of this sedimentary organic matter. These organisms are responsive to biostimulation (Meslé et al., 2013a), which could increase their cells numbers *in situ* for a potential economic methane production.

## Conflict of Interest Statement

The authors declare that the research was conducted in the absence of any commercial or financial relationships that could be construed as a potential conflict of interest.

## References

[B1] Aeckersberg F. Bak F. Widdel F. (1991). Anaerobic oxidation of saturated-hydrocarbons to CO2 by a new type of sulfate-reducing bacterium. *Arch. Microbiol.* 156 5–14. 10.1007/bf00418180

[B2] Aitken C. M. Jones D. M. Larter S. R. (2004). Anaerobic hydrocarbon biodegradation in deep subsurface oil reservoirs. *Nature* 431 291–294. 10.1038/nature02922 15372028

[B3] Atlas R. M. (1981). Microbial degradation of petroleum hydrocarbons: an environmental perspective. *Microbiol. Rev.* 45 180–209. 7012571 10.1128/mr.45.1.180-209.1981 PMC281502

[B4] Behar F. Beaumont V. Penteado H. L. D. (2001). Rock-eval 6 technology: performances and developments. *Oil Gas Sci. Technol.* 56 111–134. 10.2516/ogst:2001013

[B5] Behar F. Lorant F. Lewan M. (2008). Role of NSO compounds during primary cracking of a Type II kerogen and a Type III lignite. *Organ. Geochem.* 39 1–22. 10.1016/j.orggeochem.2007.10.007

[B6] Behar F. Vandenbroucke M. (1988). Characterization and quantification of saturates trapped inside kerogen: implications for pyrolysate composition. *Organ. Geochem.* 13 927–938. 10.1016/0146-6380(88)90246-x

[B7] Behar F. Vandenbroucke M. Pelet R. (1986). Molecular structure of kerogens and asphaltenes. *Abstr. Pap. Am. Chem. Soc.* 192 14.

[B8] Borsodi A. K. Vladar P. Cech G. Gedeon G. Beszteri B. Micsinai A. (2003). Bacterial activities in the sediment of Lake Velencei, Hungary. *Hydrobiologia* 506 721–728. 10.1023/B:HYDR.0000008586.30395.f2

[B9] Burlingame A. L. Simoneit B. R. (1969). High resolution mass spectrometry of Green River Basin formation kerogen oxidations. *Nature* 222 741–747. 10.1038/222741a0

[B10] Chou M. I. M. Kruse C. W. Lytle J. M. (1993). Organics and sulfur-containing volatiles obtained from coal pyrolysis. *Abstr. Pap. Am. Chem. Soc.* 205 655–661.

[B11] Coenye T. Vanlaere E. Samyn E. Falsen E. Larsson P. Vandamme P. (2005). *Advenella incenata* gen. nov., sp nov., a novel member of the Alcaligenaceae, isolated from various clinical samples. *Int. J. Syst. Evol. Microbiol.* 55 251–256. 10.1099/ijs.0.63267-0 15653883

[B12] Dawson K. S. Strapoc D. Huizinga B. Lidstrom U. Ashby M. Macalady J. L. (2012). Quantitative fluorescence in situ hybridization analysis of microbial consortia from a biogenic gas field in Alaska’s Cook inlet basin. *Appl. Environ. Microbiol.* 78 3599–3605. 10.1128/aem.07122-11 22427501 PMC3346356

[B13] Deley J. Segers P. Kersters K. Mannheim W. Lievens A. (1986). Intrageneric and intergeneric similarities of the *Bordetella ribosomal* nucleic-acid cistrons – proposal for a new family, Alcaligenaceae. *Int. J. Syst. Bacteriol.* 36 405–414. 10.1099/00207713-36-3-405

[B14] Deming J. W. Baross J. A. (1993). “The early diagenesis of organic matter: bacterial activity,” in *Organic Geochemistry, Principles and Applications*, eds Engel M. H. Macko S. A. (New York, NY: Plenum), 119–144.

[B15] Durand B. (2003). A history of organic geochemistry. *Oil Gas Sci. Technol.* 58 203–231. 10.2516/ogst:2003014

[B16] Emtiazi G. Shakarami H. Nahvi I. Mirdamadian S. H. (2005). Utilization of petroleum hydrocarbons by *Pseudomonas* sp. and transformed *Escherichia coli. Afr. J. Biotechnol.* 4 172–176.

[B17] Espitalié J. Laporte J. L. Madec M. Marquis F. Leplat P. Paulet J. (1977). Rapid method for source rocks characterizzation and for the determination of petroleum potential and degree of evolution. *Rev. Institut Français Pétrole* 32 23–42.

[B18] Formolo M. Martini A. Petsch S. (2008). Biodegradation of sedimentary organic matter associated with coalbed methane in the Powder River and San Juan Basins, USA. *Int. J. Coal Geol.* 76 86–97. 10.1016/j.coal.2008.03.005

[B19] Fredrickson J. K. Mckinley J. P. Bjornstad B. N. Long P. E. Ringelberg D. B. White D. C. (1997). Pore-size constraints on the activity and survival of subsurface bacteria in a late Cretaceous shale-sandstone sequence, northwestern New Mexico. *Geomicrobiol. J.* 14 183–202. 10.1080/01490459709378043

[B20] Gaspar A. Zellermann E. Lababidi S. Reece J. Schrader W. (2012). Characterization of saturates, aromatics, resins, and asphaltenes heavy crude oil fractions by atmospheric pressure laser ionization Fourier transform ion cyclotron resonance mass spectrometry. *Energy Fuels* 26 3481–3487. 10.1021/ef3001407

[B21] Grabowski A. Nercessian O. Fayolle F. Blanchet D. Jeanthon C. (2005). Microbial diversity in production waters of a low-temperature biodegraded oil reservoir. *FEMS Microbiol. Ecol.* 54 427–443. 10.1016/j.femsec.2005.05.007 16332340

[B22] Gray N. D. Sherry A. Hubert C. Dolfing J. Headt I. M. (2010). Methanogenic degradation of petroleum hydrocarbons in subsurface environments: remediation, heavy oil formation, and energy recovery. *Adv. Appl. Microbiol.* 72 137–161. 10.1016/s0065-2164(10)72005-0 20602990

[B23] Green M. S. Flanegan K. C. Gilcrease P. C. (2008). Characterization of a methanogenic consortium enriched from a coalbed methane well in the Powder River Basin, USA. *Int. J. Coal Geol.* 76 34–45. 10.1016/j.coal.2008.05.001

[B24] Guo H. Liu R. Yu Z. Zhang H. Yun J. Li Y. (2012). Pyrosequencing reveals the dominance of methylotrophic methanogenesis in a coal bed methane reservoir associated with Eastern Ordos Basin in China. *Int. J. Coal Geol.* 93 56–61. 10.1016/j.coal.2012.01.014

[B25] Haeseler F. Behar F. Garnier D. Chenet P.-Y. (2010). First stoichiometric model of oil biodegradation in natural petroleum systems Part I - The BioClass 0D approach. *Organ. Geochem.* 41 1156–1170. 10.1016/j.orggeochem.2010.05.019

[B26] Head I. M. Jones D. M. Larter S. R. (2003). Biological activity in the deep subsurface and the origin of heavy oil. *Nature* 426 344–352. 10.1038/nature02134 14628064

[B27] Hoogerheide J. C. Kocholaty W. (1938). Metabolism of the strict anaerobes (genus: *Clostridium*) II. Reduction of amino-acids with gaseous hydrogen by suspensions of Cl. sporogenes. *Biochem. J.* 32 949–957. 16746719 10.1042/bj0320949 PMC1264134

[B28] Jones D. M. Head I. M. Gray N. D. Adams J. J. Rowan A. K. Aitken C. M. (2008). Crude-oil biodegradation via methanogenesis in subsurface petroleum reservoirs. *Nature* 451 176–181. 10.1038/nature06484 18075503

[B29] Jones E. J. P. Voytek M. A. Corum M. D. Orem W. H. (2010). Stimulation of methane generation from nonproductive coal by addition of nutrients or a microbial consortium. *Appl. Environ. Microbiol.* 76 7013–7022. 10.1128/aem.00728-10 20817801 PMC2976240

[B30] Kniemeyer O. Musat F. Sievert S. M. Knittel K. Wilkes H. Blumenberg M. (2007). Anaerobic oxidation of short-chain hydrocarbons by marine sulphate-reducing bacteria. *Nature* 449 898–902. 10.1038/nature06200 17882164

[B31] Krumholz L. R. Harris S. H. Suflita J. M. (2002). Anaerobic microbial growth from components of cretaceous shales. *Geomicrobiol. J.* 19 593–602. 10.1080/01490450290098559

[B32] Krumholz L. R. Harris S. H. Tay S. T. Suflita J. M. (1999). Characterization of two subsurface H2-utilizing bacteria, *Desulfomicrobium hypogeium* sp. nov. and *Acetobacterium psammolithicum* sp. nov., and their ecological roles. *Appl. Environ. Microbiol.* 65 2300–2306. 10347005 10.1128/aem.65.6.2300-2306.1999 PMC91340

[B33] Krumholz L. R. Mckinley J. P. Ulrich F. A. Suflita J. M. (1997). Confined subsurface microbial communities in Cretaceous rock. *Nature* 386 64–66. 10.1038/386064a0

[B34] Leahy J. G. Colwell R. R. (1990). Microbial degradation of hydrocarbons in the environment. *Microbiol. Rev.* 54 305–315. 2215423 10.1128/mr.54.3.305-315.1990 PMC372779

[B35] Lee Y.-J. Romanek C. S. Mills G. L. Davis R. C. Whitman W. B. Wiegel J. (2006). *Gracilibacter thermotolerans* gen. nov., sp nov., an anaerobic, thermotolerant bacterium from a constructed wetland receiving acid sulfate water. *Int. J. Syst. Evol. Microbiol.* 56 2089–2090. 10.1099/ijs.0.64040-0 16957104

[B36] Lesueur D. (2009). The colloidal structure of bitumen: consequences on the rheology and on the mechanisms of bitumen modification. *Adv. Colloid Interface Sci.* 145 42–82. 10.1016/j.cis.2008.08.011 19012871

[B37] Lewan M. D. Ruble T. E. (2002). Comparison of petroleum generation kinetics by isothermal hydrous and nonisothermal open-system pyrolysis. *Organ. Geochem.* 33 1457–1475. 10.1016/s0146-6380(02)00182-1

[B38] Liao Y. Geng A. Huang H. (2009). The influence of biodegradation on resins and asphaltenes in the Liaohe Basin. *Organ. Geochem.* 40 312–320. 10.1016/j.orggeochem.2008.12.006

[B39] Liu Y. Whitman W. B. (2008). Metabolic, phylogenetic, and ecological diversity of the methanogenic archaea. *Ann. N. Y. Acad. Sci.* 1125 171–189. 10.1196/annals.1419.019 18378594

[B40] Magot M. Ollivier B. Patel B. K. C. (2000). Microbiology of petroleum reservoirs. *Antonie Van Leeuwenhoek* 77 103–116. 10.1023/a:1002434330514 10768470

[B41] Martini A. M. Budai J. M. Walter L. M. Schoell M. (1996). Microbial generation of economic accumulations of methane within a shallow organic-rich shale. *Nature* 383 155–158. 10.1038/383155a0

[B42] Martini A. M. Walter L. M. Ku T. C. W. Budai J. M. Mcintosh J. C. Schoell M. (2003). Microbial production and modification of gases in sedimentary basins: a geochemical case study from a Devonian shale gas play, Michigan basin. *Am. Assoc. Pet. Geol. Bull.* 87 1355–1375. 10.1306/031903200184

[B43] Martini A. M. Walter L. M. Mcintosh J. C. (2008). Identification of microbial and thermogenic gas components from Upper Devonian black shale cores, Illinois and Michigan basins. *Am. Assoc. Pet. Geol. Bull.* 92 327–339. 10.1306/10180706037

[B44] May M. E. Neihof R. A. (1982). Microbial deterioration of hydrocarbon fuels from oil-shales, coal, and petroleum. *Dev. Ind. Microbiol.* 23 495–502.

[B45] McIntosh J. Martini A. Petsch S. Huang R. Nuesslein K. (2008). Biogeochemistry of the Forest City Basin coalbed methane play. *Int. J. Coal Geol.* 76 111–118. 10.1016/j.coal.2008.03.004

[B46] Merdrignac I. Espinat D. (2007). Physicochemical characterization of petroleum fractions: the state of the art. *Oil Gas Sci. Technol.* 62 7–32. 10.2516/ogst:2007002

[B47] Meslé M. Periot C. Dromart G. Oger P. (2013a). Biostimulation to identify microbial communities involved in methane generation in shallow, kerogen-rich shales. *J. Appl. Microbiol.* 114 55–70. 10.1111/jam.12015 22979955

[B48] Meslé M. Dromart G. Oger P. (2013b). Microbial methanogenesis in subsurface oil and coal. *Res. Microbiol.* 164 959–972. 10.1016/j.resmic.2013.07.004 23872511

[B49] Milkov A. V. (2011). Worldwide distribution and significance of secondary microbial methane formed during petroleum biodegradation in conventional reservoirs. *Organ. Geochem.* 42 184–207. 10.1016/j.orggeochem.2010.12.003

[B50] Morris B. E. L. Henneberger R. Huber H. Moissl-Eichinger C. (2013). Microbial syntrophy: interaction for the common good. *FEMS Microbiol. Rev.* 37 384–406. 10.1111/1574-6976.12019 23480449

[B51] Musat F. Galushko A. Jacob J. Widdel F. Kube M. Reinhardt R. (2009). Anaerobic degradation of naphthalene and 2-methylnaphthalene by strains of marine sulfate-reducing bacteria. *Environ. Microbiol.* 11 209–219. 10.1111/j.1462-2920.2008.01756.x 18811643

[B52] Ng T. K. Weimer P. J. Zeikus J. G. (1977). Cellulolytic and physiological properties of *Clostridium thermocellum*. *Arch. Microbiol.* 114 1–7. 10.1007/bf00429622 20860

[B53] Orem W. H. Tatu C. A. Lerch H. E. Rice C. A. Bartos T. T. Bates A. L. (2007). Organic compounds in produced waters from coalbed natural gas wells in the Powder River Basin, Wyoming, USA. *Appl. Geochem.* 22 2240–2256. 10.1016/j.apgeochem.2007.04.010

[B54] Orem W. H. Voytek M. A. Jones E. J. Lerch H. E. Bates A. L. Corum M. D. (2010). Organic intermediates in the anaerobic biodegradation of coal to methane under laboratory conditions. *Organ. Geochem.* 41 997–1000. 10.1016/j.orggeochem.2010.03.005

[B55] Penner T. J. Foght J. M. Budwill K. (2010). Microbial diversity of western Canadian subsurface coal beds methanogenic coal enrichment cultures. *Int. J. Coal Geol.* 82 81–93. 10.1016/j.coal.2010.02.002

[B56] Peters K. E. Walters C. C. Moldowan J. M. (2004). *The Biomarker Guide: Biomarkers and Isotopes in Petroleum and Earth History.* Cambridge: Cambridge University Press.

[B57] Rice D. D. Claypool G. E. (1981). Generation, accumulation and resource potential of biogenic gas. *Am. Assoc. Pet. Geol. Bull.* 65 5–25.

[B58] Roling W. F. M. Head I. M. Larter S. R. (2003). The microbiology of hydrocarbon degradation in subsurface petroleum reservoirs: perspectives and prospects. *Res. Microbiol.* 154 321–328. 10.1016/s0923-2508(03)00086-x 12837507

[B59] Rueter P. Rabus R. Wilkes H. Aeckersberg F. Rainey F. A. Jannasch H. W. (1994). Anaerobic oxidation of hydrocarbons in crude-oil by new types of sulfate-reducing bacteria. *Nature* 372 455–458. 10.1038/372455a0 7984238

[B60] Sakamoto M. Benno Y. (2006). Reclassification of *Bacteroides distasonis*, *Bacteroides goldsteinii* and *Bacteroides merdae* as *Parabacteroides distasonis* gen. nov., comb. nov., *Parabacteroides goldsteinii* comb. nov. and *Parabacteroides merdae* comb. nov. *Int. J. Syst. Evol. Microbiol.* 56 1599–1605. 10.1099/ijs.0.64192-0 16825636

[B61] Sakamoto M. Kitahara M. Benno Y. (2007). *Parabacteroides johnsonii* sp nov., isolated from human faeces. *Int. J. Syst. Evol. Microbiol.* 57 293–296. 10.1099/ijs.0.64588-0 17267966

[B62] Schink B. (1997). Energetics of syntrophic cooperation in methanogenic degradation. *Microbiol. Mol. Biol. Rev.* 61 262–280. 9184013 10.1128/mmbr.61.2.262-280.1997 PMC232610

[B63] Schlegel M. E. Mcintosh J. C. Petsch S. T. Orem W. H. Jones E. J. P. Martini A. M. (2013). Extent and limits of biodegradation by in situ methanogenic consortia in shale and formation fluids. *Appl. Geochem.* 28 172–184. 10.1016/j.apgeochem.2012.10.008

[B64] Schnurer A. Schink B. Svensson B. H. (1996). *Clostridium ultunense* sp nov, a mesophilic bacterium oxidizing acetate in syntrophic association with a hydrogenotrophic methanogenic bacterium. *Int. J. Syst. Bacteriol.* 46 1145–1152. 10.1099/00207713-46-4-1145 8863449

[B65] Shurr G. W. Ridgley J. L. (2002). Unconventional shallow biogenic gas systems. *Am. Assoc. Pet. Geol. Bull.* 86 1939–1969.

[B66] Spormann A. M. Widdel F. (2000). Metabolism of alkylbenzenes, alkanes, and other hydrocarbons in anaerobic bacteria. *Biodegradation* 11 85–105. 10.1023/a:1011122631799. 11440245

[B67] Stieb M. Schink B. (1985). Anaerobic oxidation of fatty-acids by *Clostridium bryantii* sp nov, a sporeforming, obligately syntrophic bacterium. *Arch. Microbiol.* 140 387–390. 10.1007/bf00446983

[B68] Strapoc D. Mastalerz M. Dawson K. Macalady J. Callaghan A. V. Wawrik B. (2011). Biogeochemistry of microbial coal-bed methane. *Annu. Rev. Earth Planet. Sci.* 39 617–656. 10.1146/annurev-earth-040610-1333433

[B69] Strapoc D. Mastalerz M. Eble C. Schimmelmann A. (2007). Characterization of the origin of coalbed gases in southeastern Illinois Basin by compound-specific carbon and hydrogen stable isotope ratios. *Organ. Geochem.* 38 267–287. 10.1016/j.orggeochem.2006.09.005

[B70] Strapoc D. Mastalerz M. Schimmelmann A. Drobniak A. Hasenmueller N. R. (2010). Geochemical constraints on the origin and volume of gas in the New Albany Shale (Devonian-Mississippian), eastern Illinois Basin. *Am. Assoc. Pet. Geol. Bull.* 94 1713–1740. 10.1306/06301009197

[B71] Strapoc D. Mastalerz M. Schimmelmann A. Drobniak A. Hedges S. (2008). Variability of geochemical properties in a microbially dominated coalbed gas system from the eastern margin of the Illinois Basin, USA. *Int. J. Coal Geol.* 76 98–110. 10.1016/j.coal.2008.02.002

[B72] Takai K. Mormile M. R. Mckinley J. P. Brockman F. J. Holben W. E. Kovacik W. P. (2003). Shifts in archaeal communities associated with lithological and geochemical variations in subsurface Cretaceous rock. *Environ. Microbiol.* 5 309–320. 10.1046/j.1462-2920.2003.00421.x 12662178

[B73] Tissot B. (1977). How petrolum forms. *Recherche* 8 326–334.

[B74] Tissot B. Durand B. Espitali J. Combaz A. (1974). Influence of nature and diagenesis of organic-matter in formation of petroleum. *Am. Assoc. Pet. Geol. Bull.* 58 499–506.

[B75] Tissot B. P. Welte D. H. (1984). *Petroleum Formation and Occurrence.* Berlin: Springer-Verlag 10.1007/978-3-642-87813-8

[B76] Uroz S. Buee M. Murat C. Frey-Klett P. Martin F. (2010). Pyrosequencing reveals a contrasted bacterial diversity between oak rhizosphere and surrounding soil. *Environ. Microbiol. Rep.* 2 281–288. 10.1111/j.1758-2229.2009.00117.x 23766079

[B77] Vandecasteele J.-P. (2005). *Microbiologie Pétrolière. Concepts, Implications Environnementales, Applications Industrielles.* Paris: IFP Publications.

[B78] Vandenbroucke M. (2003). Kerogen: from types to models of chemical structure. *Oil Gas Sci. Technol.* 58 243–269. 10.2516/ogst:2003016

[B79] Vandenbroucke M. Largeau C. (2007). Kerogen origin, evolution and structure. *Organ. Geochem.* 38 719–833. 10.1016/j.orggeochem.2007.01.001

[B80] Walker J. D. Petrakis L. Colwell R. R. (1976). Comparison of biodegradability of crude and fuel oils. *Can. J. Microbiol.* 22 598–602. 10.1139/m76-089 1260549

[B81] Warwick P. D. Breland F. C. Jr. Hackley P. C. (2008). Biogenic origin of coalbed gas in the northern Gulf of Mexico Coastal Plain, USA. *Int. J. Coal Geol.* 76 119–137. 10.1016/j.coal.2008.05.009

[B82] Wawrik B. Mendivelso M. Parisi V. A. Suflita J. M. Davidova I. A. Marks C. R. (2012). Field and laboratory studies on the bioconversion of coal to methane in the San Juan Basin. *FEMS Microbiol. Ecol.* 81 26–42. 10.1111/j.1574-6941.2011.01272.x 22146015

[B83] Whiticar M. J. (1999). Carbon and hydrogen isotope systematics of bacterial formation and oxidation of methane. *Chem. Geol.* 161 291–314. 10.1016/s0009-2541(99)00092-3

[B84] Widdel F. Rabus R. (2001). Anaerobic biodegradation of saturated and aromatic hydrocarbons. *Curr. Opin. Biotechnol.* 12 259–276. 10.1016/s0958-1669(00)00209-3 11404104

[B85] Yen T. F. (1976). Structural aspects of organic components in oil shales. *Oil Shale Dev. Pet. Sci.* 5 120–148.

[B86] Zakrzewski M. Goesmann A. Jaenicke S. Juenemann S. Eikmeyer F. Szczepanowski R. (2012). Profiling of the metabolically active community from a production-scale biogas plant by means of high-throughput metatranscriptome sequencing. *J. Biotechnol.* 158 248–258. 10.1016/j.jbiotec.2012.01.020 22342600

[B87] Zengler K. Richnow H. H. Rossello-Mora R. Michaelis W. Widdel F. (1999). Methane formation from long-chain alkanes by anaerobic microorganisms. *Nature* 401 266–269. 10.1038/45777 10499582

